# Molecular epidemiological characteristics of carbapenem-resistant *Klebsiella pneumoniae* among children in China

**DOI:** 10.1186/s13568-022-01437-3

**Published:** 2022-07-12

**Authors:** Zhengjiang Jin, Zhenhui Wang, Lin Gong, Lu Yi, Nian Liu, Lan Luo, Wenting Gong

**Affiliations:** 1 Department of Clinical Laboratory, Maternal and Child Health Hospital of Hubei Province, Wuhan, 430070 China; 2 Department of Disinfection and Pest Control, Wuhan Center for Disease Control & Prevention, Wuhan, 430000 China; 3 Department of Child Health, Maternal and Child Health Hospital of Hubei Province, Wuhan, 430070 China; 4 Department of Pharmacy, Maternal and Child Health Hospital of Hubei Province, Wuhan, 430070 China

**Keywords:** *Klebsiella pneumoniae*, Infection, Carbapenem resistance, Virulence, Disinfectant resistance

## Abstract

*Klebsiella pneumoniae* infection and antimicrobial resistance among children are major concerns. The occurrence of hypervirulent *K. pneumoniae* (hvKp) infections is gradually increasing worldwide, and disinfectant resistance is also being reported. Carbapenem- and disinfectant-resistant hvKp infection has made clinical treatment and nosocomial infection control among children increasingly challenging. In this study, whole-genome sequencing was conducted among 34 Carba NP-positive carbapenem-resistant *K. pneumoniae* (CRKP) strains, and the distribution of antibiotic resistance genes, virulence genes and disinfectant resistance genes was determined. Eleven distinct STs were identified, and most of them were ST11 (58.8%). Among the carbapenem resistance genes, *KPC-2* was predominant (61.8%), followed by *NDM-1* (26.5%) and *IPM-4* (11.8%), and no other carbapenemase genes were found. Twelve virulence genes were investigated. All 34 CRKP strains carried the following virulence genes: *rcsA/B, entA, fimA/H* and *mrkA/D.* The gene *iucB* was present in only 3 (8.9%) CRKP strains. The positive detection rates of the *iroN* and *ybtA* genes were 94.1% and 64.7%, respectively. None of the strains was found to carry the *rmpA* and *iroB genes.* Two disinfectant resistance genes were investigated in this study. Twenty-one (61.8%) strains carried both the *qacE and cepA* disinfectant resistance genes, 13 (38.2%) CRKP strains carried only the *cepA* gene, and no strains with only the *qacE* gene was detected. The correlations among virulence, drug resistance and disinfectant tolerance showed that the virulence and disinfectant resistance genes were distinct among several types of carbapenemase-producing CRKP strains.

## Introduction

With the widespread use of carbapenems worldwide, corresponding carbapenem-resistant *Enterobacterales* (CRE) strains have emerged (Wyres and Holt 2018). The threat posed by CRE is enormous because carbapenems have traditionally been used to treat infections caused by *Enterobacterales* producing extended-spectrum β-lactamases (ESBLs), and are still considered a last line of defence against *Enterobacterales* (Zheng et al. 2018). *Klebsiella pneumoniae* is a prominent member of the CRE family that is prevalent worldwide and has a high mortality rate. According to the data from the 2018 China antimicrobial surveillance network (CHINET), *K. pneumoniae* was second only to *Escherichia coli* in the number of isolated Gram-negative bacilli (Hu et al. 2019). In addition, 2018 CHINET data also showed that the resistance rates of *K. pneumoniae* to meropenem and imipenem were 26.3% and 25%, respectively. However, in some children’s hospitals, the rates ranged from 32.1% to 45.5%, which means that the resistance of *K. pneumoniae* to carbapenems in children should be given serious attention (Zhang et al. 2018).

The development of carbapenem resistance mechanisms in *K. pneumoniae* tends to occur when they acquire plasmids containing multiple antibiotic resistance genes, the most common of which are ESBL genes (such as *CTX-M*, *TEM*, *SHV,* and *OXA*); another pathway involves their acquiring genes encoding carbapenemases ( such as *KPC*, *NDM* and *VIM*). In recent years, reports on carbapenem-resistant *Klebsiella pneumoniae* (CRKP) infection have gradually increased, but the situation varies by region (Shon et al. 2013; Fu et al. 2019; Luo et al. 2021).

Currently, most *K. pneumoniae* infections are caused by the classic *K. pneumoniae* (cKp) (Russo et al. 2019). However, in recent years, cases of highly virulent *K. pneumoniae* (hvKp) infection increased worldwide, which has aroused great concern (Beyrouthy et al. 2020; Yang et al. 2020; Chen and Chen 2021). Highly virulent strains are not only highly pathogenic but may also may be resistant to antibiotics and disinfectants. However, there are few related research reports (Soto et al. 2020; Gharieb et al. 2022). To the best of our knowledge and according to a survey of the literature, there have been few studies on the prevalence of virulence genes and disinfectant resistance genes in clinical CRKP isolates from children in China (Pereira et al. 2016). Therefore, this study aimed to investigate the prevalence of the virulence factors, carbapenemases, and disinfectant resistance genes of *K. pneumoniae* strains isolated from clinical specimens collected from children and to evaluate the associations among potential virulence factors, carbapenem resistance, and disinfectant tolerance.

## Materials and methods

### Study design

This was a retrospective study performed in our clinical setting, which is a tertiary care centre for women and children in Wuhan city in central China, caring on average for 6000 children hospitalized annually. Hospitalized patients with *K. pneumoniae -*positive cultures from January 2019 to December 2021 were included in this investigation. If the laboratory findings (CRP > 10 mg/L or PCT > 0.50 μg/L) were compatible with a clinical infection associated with isolation of *K. pneumoniae* in a relevant biologic sample, the patient was defined as infected. Only *K. pneumoniae* strains cultured from infected children from January 2019 to December 2021 were collected and kept frozen in the hospital laboratory department, while duplicate strains from the same patient were excluded. These strains were thawed and cultured for microbiological analysis.

### Microbiological analysis

*K. pneumoniae* strains were identified by matrix-assisted laser desorption ionization time-of-flight mass spectrometry (MALDI-TOF MS; Bruker Daltonics, Germany), and antimicrobial susceptibility assays were performed by the AST GN13 panel on a VITEK 2 COMPACT instrument (BioMerieux, France), according to the manufacturer’s instructions. After recovery, the carbapenem-resistant strains were cultured on blood agar plates, and the Carba NP test was performed according to the guidelines of the Clinical and Laboratory Standards Institute (CLSI) (2019).

### Whole-genome sequencing and phylogenetic analysis

For Carba NP -positive CRKP strains, whole-genome sequencing was conducted, and the distribution of antibiotic resistance genes, virulence genes, and disinfectant resistance genes was determined based on the Center for Genomic Epidemiology (CGE) database and Virulence Factors Database (VFDB) foe pathogenic bacteria. At the same time, the STs of the strains were determined by CGE database comparison (Luo et al. 2021).

GTDB-Tk (1.3.0) was used to perform multiple sequence alignment of bacterial sequences obtained in this study and other CRKP genome sequences published in the NCBI database. The results of the multiple sequence alignment were used to construct phylogenetic trees using the maximum likelihood method in MEGA11 software. The tree construction results were visualized using the R language ggtree package (Wyres et al. 2020).

### Statistical analysis

Data were first entered into Excel and then transferred and analysed using SPSS 21.0. Categorical variables were compared using the chi-squared test or Fisher’s exact test. A P value < 0.05 was considered to indicate statistically significance. P values for multiple testing were corrected by the false discovery rate (FDR). The Benjamini and Hochberg method was used to calculate the FDR. WHONET 5.6 was used to analyse the antimicrobial resistance rates and resistance patterns.

## Results

### K. pneumoniae infection cases

From January 2019 to December 2021, a total of 6,014 children who had a positive culture were hospitalized in our clinical setting, and among them, 329 (5.5%) were positive for *K. pneumoniae.* According to the laboratory findings (CRP > 10 mg/L or PCT > 0.50 μg/L), among the 329 patients, there were 230 (69.9%) *K. pneumoniae* infection cases, 52.9% of which were associated with isolates from respiratory samples, and 99 (30.1%) cases associated with colonization. Among the infected patients, 230 harboured unique *K. pneumoniae* strains, and their distribution is shown in Table 1. The sex and ward distributions of the *K. pneumoniae* infection cases and colonization cases were not significantly different (p > 0.05), except for the sampled specimen types (p < 0.05).

**Table 1:** Distribution of sex,department and specimen between infection cases and colonized cases caused by *K.pneumoniae*

Project	*K.pneumoniae* infection cases n(%)	*K.pneumoniae* colonized cases n(%)	P value
Sex			
Female	97(42.2)	41(41.4)	0.898
Male	133(57.8)	58(58.6)	
Ward			
Neonatology	140(60.9)	59(59.6)	0.828
Pediatrics	90(39.1)	40(40.4)	
Specimen			
Sputum	137(59.6)	87(87.9)	< 0.001
Urine	37(16.1)	3(3.0)	
Blood	17(7.4)	0	
Catheter	16(7.0)	5(5.1)	
Broncho-alveolar lavage	8(3.5)	0	
Gastric fluid	6(2.6)	0	
Others	9(3.9)	4(4.0)	

*n*  number,%  percentage

### Antibiotic susceptibility and resistance of strains

According to the carbapenem antibiotic susceptibility test results for all 230 K*. pneumoniae* strains, 59 (25.7%) was found to be in the CRKP group, and 171 (74.3%) were in the non-CRKP group. The drug resistance of the CRKP and non-CRKP groups to various antibiotics is shown in Table 2. The resistance rates of all the strains in the CRKP group to all the tested antibiotics were higher than those of the strains in the non-CRKP group (*p* ≤ *0.001*), except for trimethoprim/sulfamethoxazole (*p* > *0.05*).

**Table 2:** Antibiotic resistance patterns between CRKP and non-CRKP strains

Antibiotics	CRKP strains n(%)	non-CRKP strains n(%)	P value
*Ampicillin*	59(100)	144(84.3)	0.001
*Ampicillin/Sulbactam*	59(100)	54(31.6)	< 0.001
*Piperacillin/Tazobactam*	49(82.9)	2(1.2)	< 0.001
*Ceftazidime*	54(91.4)	18(10.5)	< 0.001
*Ceftriaxone*	59(100)	49(28.7)	< 0.001
*Cefepime*	50(84.6)	19(11.1)	< 0.001
*Cefotetan*	42(71.9)	1(0.6)	< 0.001
*Aztreonam*	46(78.1)	33(19.3)	< 0.001
*Amikacin*	25(42.9)	1(0.6)	< 0.001
*Gentamicin*	13(21.9)	12(7.0)	0.001
*Tobramycin*	28(46.9)	5(2.9)	< 0.001
*Ciprofloxacin*	33(56.2)	10(5.8)	< 0.001
*Levofloxacin*	34(57.1)	5(2.9)	< 0.001
*Trimethoprim/Sulfamethoxazole*	14(22.9)	25(14.6)	0.108

*n * number of resistant isolates,% percentage of resistant isolates

### Carba NP test and whole-genome sequencing results

The 59 CRKP strains isolated from these *K. pneumoniae* infection cases were kept frozen in the microbiology laboratory in our hospital. After resuscitation, the strains were inoculated on blood agar plates, and the Carba NP test was performed. The results showed that 34 strains (57.6%) were Carba NP positive, 17 (28.8%) strains were Carba NP negative, and 8 (13.6%) strains had inconclusive results. The 34 Carba NP-positive strains were subjected to whole-genome sequencing. These whole-genome sequencing results have been deposited at DDBJ/ENA/GenBank under the accession numbers JALPZL000000000, JALYAY000000000, JALYAZ000000000, JALYBA/B/C/D/E/F/G/H/I/J/K/L/M/N/O/P000000000, JAMCAJ/K/L/M000000000, and JAMSHQ000000000. Based on the whole-genome sequencing results, 11 distinct STs were identified, and using database comparison, most of them were found to be ST11 (58.8%). The distribution of antibiotic resistance genes is presented in Table 3. Of the 34 patients infected with the various CRKP isolates, 22 (64.7%) were male, 28 (82.3%) were from the pediatric unit, and the average age was 5.41 months (the standard deviation (SD), was 9.79 months). Among all 34 CRKP strains, the most frequent source of isolation was sputum specimens, accounting for 41.2%, followed by catheters and urine, both accounting for 20.6%. The major drug resistance genes coding for resistance to carbapenem were identified in the 34 investigated CRKP isolates. Among the carbapenem resistance genes, *KPC-2* was predominant (61.8%), followed by *NDM-1* (26.5%) and *IPM-4* (11.8%),and no other carbapenemase genes were found.

**Table 3:** Distribution of antibiotic resistance genes, virulence genes and disinfectant resistance genes among 34 CRKP strains

Isolate no	Patient gender	Age	Ward	Specimen	ST type	Carba NP	Drug resistance genes	Virulence genes	Disinfectant resistance genes
Kpn1	Female	2d	Neonatology	Blood	464	+	*IMP-4*	*rcsA/B,iroN,entA,fimA/H,mrkA/D*	*cepA*
Kpn2	Female	1y	Pediatrics	Urine	11	+	*KPC-2*	*rcsA/B,iroN,ybtA,entA,fimA/H,mrkA/D*	*qacE,cepA*
Kpn3	Male	16d	Neonatology	Sputum	11	+	*KPC-2*	*rcsA/B,iroN,ybtA,entA,fimA/H,mrkA/D*	*qacE,cepA*
Kpn4	Male	30d	Neonatology	Sputum	11	+	*KPC-2*	*rcsA/B,iroN,ybtA,entA,fimA/H,mrkA/D*	*qacE,cepA*
Kpn5	Male	5d	Neonatology	Umbilicus	2407	+	*IMP-4*	*rcsA/B,iroN,entA,fimA/H,mrkA/D*	*cepA*
Kpn6	Female	1m	Pediatrics	Sputum	2407	+	*IMP-4*	*rcsA/B,iroN,entA,fimA/H,mrkA/D*	*cepA*
Kpn7	Male	5m	Pediatrics	Urine	11	+	*KPC-2*	*rcsA/B,iroN,ybtA,entA,fimA/H,mrkA/D*	*qacE,cepA*
Kpn8	Female	2m	Pediatrics	Sputum	20	+	*NDM-1*	*rcsA/B,iroN,entA,fimA/H,mrkA/D*	*cepA*
Kpn9	Female	2m	Pediatrics	Sputum	20	+	*NDM-1*	*rcsA/B,iroN,entA,fimA/H,mrkA/D*	*cepA*
Kpn10	Male	3m	Pediatrics	Sputum	11	+	*KPC-2*	*rcsA/B,iroN,ybtA,entA,fimA/H,mrkA/D*	*qacE,cepA*
Kpn11	Male	4y	Pediatrics	Broncho-alveolar lavage	11	+	*KPC-2*	*rcsA/B,iroN,ybtA,entA,fimA/H,mrkA/D*	*qacE,cepA*
Kpn12	Male	2m	Pediatrics	Urine	11	+	*KPC-2*	*rcsA/B,iroN,ybtA,entA,fimA/H,mrkA/D*	*qacE,cepA*
Kpn13	Female	6m	Pediatrics	Catheter	11	+	*KPC-2*	*rcsA/B,iroN,ybtA,entA,fimA/H,mrkA/D*	*qacE,cepA*
Kpn14	Female	2m	Pediatrics	Urine	11	+	*KPC-2*	*rcsA/B,iroN,ybtA,entA,fimA/H,mrkA/D*	*qacE,cepA*
Kpn15	Male	1m	Pediatrics	Urine	1681	+	*NDM-1*	*rcsA/B,iroN,entA,fimA/H,mrkA/D*	*cepA*
Kpn16	Male	7m	Pediatrics	Broncho-alveolar lavage	1308	+	*IMP-4*	*rcsA/B,iroN,entA,fimA/H,mrkA/D*	*cepA*
Kpn17	Male	1m	Pediatrics	Sputum	1681	+	*NDM-1*	*rcsA/B,iroN,entA,fimA/H,mrkA/D*	*cepA*
Kpn18	Female	4m	Pediatrics	Catheter	11	+	*KPC-2*	*rcsA/B,iroN,ybtA,entA,fimA/H,mrkA/D*	*cepA*
Kpn19	Male	7m	Pediatrics	Sputum	11	+	*KPC-2*	*rcsA/B,iroN,ybtA,entA,fimA/H,mrkA/D*	*qacE,cepA*
Kpn20	Male	7d	Neonatology	Sputum	198	+	*NDM-1*	*rcsA/B,iroN,ybtA,entA,fimA/H,mrkA/D*	*qacE,cepA*
Kpn21	Male	8m	Pediatrics	Sputum	11	+	*KPC-2*	*rcsA/B,iucB,iroN,entA,fimA/H,mrkA/D*	*qacE,cepA*
Kpn22	Male	3m	Pediatrics	Catheter	414	+	*NDM-1*	*rcsA/B,ybtA,entA,fimA/H,mrkA/D*	*qacE,cepA*
Kpn23	Female	4m	Pediatrics	Catheter	11	+	*KPC-2*	*rcsA/B,iroN,ybtA,entA,fimA/H,mrkA/D*	*cepA*
Kpn24	Male	3y	Pediatrics	Catheter	11	+	*KPC-2*	*rcsA/B,iroN,ybtA,entA,fimA/H,mrkA/D*	*qacE,cepA*
Kpn25	Female	2m	Pediatrics	Sputum	11	+	*KPC-2*	*rcsA/B,iroN,ybtA,entA,fimA/H,mrkA/D*	*qacE,cepA*
Kpn26	Male	4m	Pediatrics	Sputum	198	+	*NDM-1*	*rcsA/B,iroN,entA,fimA/H,mrkA/D*	*cepA*
Kpn27	Female	2m	Pediatrics	Broncho-alveolar lavage	3155	+	*NDM-1*	*rcsA/B,iucB,iroN,ybtA,entA,fimA/H,mrkA/D*	*qacE,cepA*
Kpn28	Female	2m	Pediatrics	Sputum	11	+	*KPC-2*	*rcsA/B,iroN,ybtA,entA,fimA/H,mrkA/D*	*qacE,cepA*
Kpn29	Male	1m	Pediatrics	Broncho-alveolar lavage	11	+	*KPC-2*	*rcsA/B,iroN,ybtA,entA,fimA/H,mrkA/D*	*qacE,cepA*
Kpn30	Male	4m	Pediatrics	Catheter	11	+	*KPC-2*	*rcsA/B,iroN,ybtA,entA,fimA/H,mrkA/D*	*qacE,cepA*
Kpn31	Male	1m	Pediatrics	Catheter	1770	+	*KPC-2*	*rcsA/B,entA,fimA/H,mrkA/D*	*cepA*
Kpn32	Male	8m	Pediatrics	Urine	11	+	*KPC-2*	*rcsA/B,iroN,ybtA,entA,fimA/H,mrkA/D*	*qacE,cepA*
Kpn33	Male	25d	Neonatology	Sputum	313	+	*NDM-1*	*rcsA/B,iucB,iroN,entA,fimA/H,mrkA/D*	*cepA*
Kpn34	Male	3m	Pediatrics	Urine	11	+	*KPC-2*	*rcsA/B,iroN,ybtA,entA,fimA/H,mrkA/D*	*qacE,cepA*

*d* days, *m* months, *y* years, *ST* strain + positive;

The results for virulence genes are presented in Table 3, and 12 virulence genes were investigated in a total of 34 CRKP isolates, including capsule synthesis-related genes (*rmpA, rcsA/B*), fimbriae synthesis-related genes (*fimA/H, mrkA/D*), and iron uptake-related genes (*iucB, iroB, iroN, ybtA, entA*). All 34 CRKP strains carried the following virulence genes: *rcsA/B, entA,fimA/H* and *mrkA/D.* The gene *iucB* was present in only 3 (8.9%) CRKP strains. The positive detection rates for the *iroN* and *ybtA* genes were 94.1% and 64.7%, respectively. None of the 34 CRKP isolates were found to carry *rmpA* or *iroB.*

The results for disinfectant resistance genes are presented in Table 3, and 2 disinfectant resistance genes were investigated in the 34 CRKP isolates. Twenty-one (61.8%) CRKP strains carried both the *qacE and cepA* disinfectant resistance genes, 13 (38.2%) CRKP strains carried only the *cepA* gene, and no strains with only the *qacE* gene was detected.

### Phylogenetic relationships among isolates

**Fig. 1:**
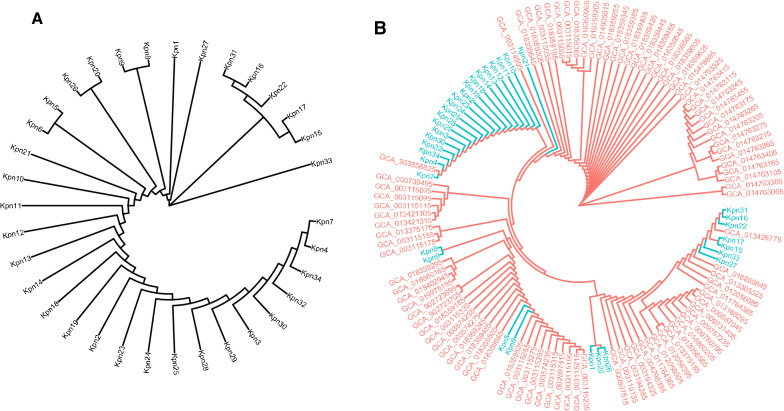
Phylogenetic relationships between CRKP isolates. **A** The phylogenetic relationships between 34 CRKP isolates included in this study. **B** The phylogenetic relationships between all 128 CRKP isolates, 34 CRKP isolates included in this study (Kpn1-Kpn34, green font), 94 CRKP isolates from the NCBI database (GCA_, red font). GCA_903856825 was submitted by Zhejiang University, China

The phylogenetic relationships among the 34 CRKP isolates included in this study are presented in Fig. 1A. Other CRKP genome searches were performed using the NCBI Biosample subdatabase. Using the search keywords “carbapenem resistant” and “Klebsiella pneumoniae”, 882 search results were obtained, and 94 strains were retained in the Genome sub-database. These data were submitted by different countries around the world, including China. The phylogenetic relationships among all 128 CRKP isolates are presented in Fig. 1B. According to the phylogenetic distances, the 34 CRKP strains were divided into three groups, and had a certain phylogenetic relationship with strains obtained from the NCBI database.

### Associations among virulence,drug resistance and disinfectant tolerance

The correlations among virulence, drug resistance and disinfectant tolerance are presented in Table 4. The virulence and disinfectant resistance genes were carried distinct among several types of carbapenemase-producing CRKP strains. Among the *KPC-2-*producing isolates, most strains carried both the *iroN* and *ybtA* virulence genes*,* followed by strains carrying only one of these genes. However, in *NDM-1-*producing strains and *IPM-4*-producing strains, there was a slight difference, the carriage rate of both the *iroN* and *ybtA* genes was very low (22.2% and 0), and there was a statistically significant difference among the three groups (*p* < 0.001). Among disinfectant resistance genes, all 4 *IPM-4*-producing isolates carried only the *cepA* gene, but in *KPC-2-*producing strains and *NDM-1-*producing strains*,* there were* 3* and* 5* genes*,* respectively, and there was a statistically significant difference (*p* < 0.001). A total of 85.7% of *KPC-2-*producing strains and 44.4% of *NDM-1-*producing strains carried both the *qacE* and *cepA* genes, but none of the *IPM-4*-producing isolates carried these genes, and there was a statistically significant difference (*p* < 0.001).

**Table 4:** Association between virulence,drug resistance and disinfectant tolerance

Characteristics	KPC-2 isolates (n = 21)	NDM-1 isolates (n = 9)	IPM-4 isolates (n = 4)	P value*
Virulence genes				
*rcsA/B* + *entA* + *fimA/H* + *mrkA/D*	21(100)	9(100)	4(100)	-
* rcsA/B* + *entA* + *fimA/H* + *mrkA/D* + *iroN*	20(95.2)	8(88.9)	4(100)	0.690
* rcsA/B* + *entA* + *fimA/H* + *mrkA/D* + *ybtA*	0	1(11.1)	0	0.304
* rcsA/B* + *entA* + *fimA/H* + *mrkA/D* + *iroN* + *ybtA*	19(90.5)	2(22.2)	0	< 0.001
* rcsA/B* + *entA* + *fimA/H* + *mrkA/D* + *iroN* + *iucB*	1(4.8)	2(22.2)	0	0.304
Disinfectant resistance genes				
* cepA*	3(14.3)	5(55.6)	4(100)	0.003
* qacE* + *cepA*	18(85.7)	4(44.4)	0	0.003

*n*  number of positive isolates, % percentage of positive isolates

*P value had been corrected by false discovery rate

## Discussion

*K. pneumoniae* is a Gram-negative, encapsulated *Enterobacterales* species that is widely present in the environment and is parasitic on the skin, and in the nasopharynx and intestinal tract of humans. It is an opportunistic pathogen, that often infects immunocompromised persons. In recent years, increasing attention has been given to *K. pneumoniae*, focusing on its high rate of drug resistance and high virulence. However, disinfectant resistance has received little attention among *K. pneumoniae* strains (Candan and Aksöz 2015; Aygun et al. 2019; Surgers et al. 2019).

Antimicrobial resistance has emerged as one of the greatest threats to public health, and rising resistance to carbapenems is of particular concern due to the lack of effective and safe alternative treatment options. The results of this study showed that the resistance rate of *K. pneumoniae* to carbapenems in paediatric patients was 25.7%, which was lower than that reported by CHINET in 2018 and may be related to regional differences (Hu et al. 2019). The resistance of *K. pneumoniae* to carbapenems is mediated by different resistance mechanisms, including the production of carbapenemase, the change in porins and the increase in efflux pump activity, the most important of which is the production of carbapenemase (Kopotsa et al. 2019; Hansen 2021; Lan et al. 2021).

Carbapenemase is a β-lactamase that hydrolyses carbapenem antibiotics such as ertapenem, imipenem, and meropenem. Usually, carbapenemase can also hydrolyse β-lactam antibiotics, such as penicillins, β-lactams, β-lactamase inhibitor compound preparations, and cephalosporins (Van Duin and Doi 2017). In general, carbapenemase-producing *K. pneumoniae* strains exhibit resistance to all current β-lactam drugs. Consequently, the results of this study showed that the resistance rate of CRKP strains to commonly used antibiotics was generally higher than that of non-CRKP strains (p < 0.05). Carbapenemase production is the main reason for carbapenem resistance among *K. pneumoniae* strains. According to differences in their molecular structures, carbapenemases are classified into three classes, namely, A, B and D β-lactamases in the Ambler classification system, and are encoded by the *bla* gene. Since the identification of IMP-1 from *K. pneumoniae*, various carbapenemases have been discovered successively, such as VIM-1, NDM-1, OXA-48, and KPC-2. Among them, the KPC enzyme has become the prevalent carbapenemase and can be transmitted rapidly by plasmids (Chen et al. 2014; Tamma and Simner 2018; Hansen 2021). In this study, the KPC-2 carbapenemase accounted for 61.8% of the carbapenem resistance and was the most dominant type, which was consistent with related reports (Han et al. 2020). This was followed by the NDM-1 type and IPM-4 type, accounting for 26.5% and 11.8%, respectively; both NDM-1 and IPM-4 are metallo-β-lactamases (MBLs). Since NDM-1 was first discovered in 2008 in a Swedish patient in India with *K. pneumoniae* infection, it has spread worldwide. In China, since the first NDM-1-positive isolate was found in Hunan Province in 2012, NDM-1-producing *K. pneumoniae* has emerged in numerous areas of China. According to the literature, it is especially common in children and is significantly associated with mortality, which can be as high as 20–64% and deserves urgent attention (Ho et al. 2012; Yu et al. 2017; Huang et al. 2018).

In this study, sequence typing was performed by database alignment after whole-genome sequencing. Eleven STs were detected after analysing all 34 CRKP clinical isolates, among which ST11 accounted for 58.8%, and all of them were KPC-2-producing strains, which was consistent with the relevant literature reports (Hansen 2021). Among the NDM-1-producing strains, the STs were ST20 (22.2%), ST198 (22.2%), ST313 (11.1%), ST414 (11.1%), ST1681 (22.2%), and ST3155 (11.1%). However, among the IPM-4-producing strains, the STs were ST464 (25.0%), ST1308 (25.0%) and ST2407 (50.0%). The phylogenetic relationships among CRKP isolates were examined in this study. The results showed that the 34 CRKP strains was divided into three groups, however, in terms of sex, age, ward, drug resistance gene, virulence gene, disinfectant resistance gene and other aspects, the group did not show obvious aggregation. According to the phylogenetic distances of all 128 CRKP isolates, one genome (Assembly: GCA_903856825, GenBank: CAIODC000000000) was submitted by Zhejiang University China and had shared high homology with the Kpn7 genome (GenBank: JALYAZ000000000) in our sequencing results. Therefore more attention should be given to the prevention of the spread of drug-resistant strains among different regions (Price et al. 2022).

The first case of hvKP infection was reported in 1986, and since then, reports of hvKP infection have gradually increased worldwide (Catalán-Nájera et al. 2017). However, clinical microbiology laboratories cannot accurately distinguish cKP and hvKP, which hinders the timely treatment of patients with hvKP infection. Therefore, the detection of hvKP virulence is of vital importance. Early diagnosis and prompt treatment can improve the prognosis of infections caused by these strains; however, there remains a lack of exact molecular diagnostic criteria and specific molecular markers. At present, the known genes related to the virulence of *K. pneumoniae* include genes related to capsular polysaccharide synthesis and synthesis regulation, genes related to fimbriae synthesis, and genes related to the iron uptake system (Brisse et al. 2009; Li et al. 2014; Clegg and Murphy 2016). A total of 12 virulence genes were detected in this study, including *rmpA*, *rcsA/B* (capsule synthesis regulation related genes), *fimA/H, mrkA/D* (fimbriae synthesis related genes), *iucB, iroB, iroN, ybtA,* and *entA* (iron uptake related genes). Capsular polysaccharide is an important virulence factor of *K. pneumoniae*, which helps bacteria escape immunity by resisting macrophage phagocytosis, inhibiting the early inflammatory response, resisting the action of antimicrobial peptides, and inhibiting dendritic cell maturation. Increased capsular production is associated with the hypervirulence phenotype of *K. pneumoniae*, and the *rmpA* and *rcsA/B* genes are involved in regulating and affecting the synthesis of capsular polysaccharides (Brisse et al. 2009; Peng et al. 2018). In this study, the carriage rate of the *rcsA/B* gene was 100%, and there was no difference among multiple types of carbapenemase-producing strains. Although some studies have used the *rmpA* gene as the most accurate molecular marker of hvKP, no strains carrying the *rmpA* gene were detected in this study. Fimbriae contribute to bacterial colonization and biofilm formation, and T1P and T3P are the main fimbriae of *K. pneumoniae*. T1P is encoded by the *fimA* and *fimH* genes, which can mediate the binding of bacteria to mannose-containing receptors on host cells so that the bacteria can colonize the urogenital tract, respiratory tract, and intestinal tract. T3P is encoded by the *mrkA* and *mrkD* genes and adheres to endothelial cells and epithelial cells of the respiratory tract and urinary tract (Gerlach et al. 1989). In this study, the carriage rates of the *fimA/H* and *mrkA/D* genes were both 100%, and there was no difference between several types of carbapenemase-producing strains. The iron uptake system is an important molecular mechanism of bacterial virulence. *K. pneumoniae* has four kinds of iron carriers, namely enterobactin, aerobactin, salmochelin, and yersiniabactin*,* among which aerobactin, salmochelin, and yersiniabactin are the most common in hvKP (Lawlor et al. 2007; Hsieh et al. 2008; Russo et al. 2015; Lam et al. 2018). Enterobactin is encoded by the *entABCDE* gene. In this study, the carriage rate of the *entA* gene was 100%, and there was no difference among several types of carbapenemase-producing strains. Aerobactin is encoded by *iucABCD* and is less highly expressed in cKp, but it is usually present in hvKP. In this study, the *iucB* gene was detected in only 3 strains of CRKP, with a positivity rate of 8.9%, of which 1 strain was a KPC-2 producing strain and 2 were NDM-1 producing strains. Salmochelin is encoded by the *iroBCDN* gene, and yersiniabactin is synthesized by the *ybt* gene. In this study, the *iroB* gene was not detected, and the positivity rate of the *iroN* gene, which was distributed in the KPC-2, NDM-1, and IPM-4 strains, was 94.1%. The positivity rate of the *ybtA* gene, distributed in KPC-2 and NDM-1 strains, was 64.7%, but this gene was not detected in IPM-4 strains, while 19 KPC-2 strains and 2 NDM-1 strains carried *iroN* and *ybtA* at the same time, which was statistically significant (p < 0.001).

The correct use of disinfectants plays a key role in the prevention and control of nosocomial infections and is the basis of any effective plan for the prevention and control of nosocomial infections. Quaternary ammonium compounds are also widely used as disinfectants in hospitals, and resistance genes (*qac*) for these compounds are widespread. *qacE* was originally discovered in a plasmid of *Klebsiella aerogenes*. The mechanism of *qac* gene-mediated resistance to quaternary ammonium compounds in *K. pneumoniae* involves thermodynamics-dependent efflux (Azadpour et al. 2015). Chlorhexidine is a cationic preservative and a biguanide compound that can be used as a topical agent, has activity against a variety of bacteria and is widely used. Chlorhexidine cationic components react with anionic microbial cell surfaces to kill bacteria through membrane damage and intracellular damage. Gram-negative bacteria in hospitals, including *K. pneumoniae*, can develop resistance to chlorhexidine. The mechanism of chlorhexidine resistance in Gram-negative bacteria is unclear, but an association between the *cepA* gene and chlorhexidine resistance has been found in *K. pneumoniae* (Abuzaid and Amyes 2015; Azadpour et al. 2015). In this study, there were differences in the distribution of the *qacE* gene and *cepA* gene in different carbapenemase producing *K. pneumoniae* strains (p < 0.001). In all 34 CRKP strains, the *cepA* gene was widespread, and the carriage rate was 100%, similar to reports in the literature, but no strain carrying only the *qacE* gene was found in this study. A total of 85.7% of KPC-2-producing strains and 44.4% of NDM-1-producing strains carried both the *qacE* and *cepA* genes, and all four IPM-4-producing strains carried only the *cepA* gene. It has been reported that integrons, as mobile elements, play a vital role in the molecular mechanism of drug resistance and disinfectant resistance. Carbapenemase genes and disinfectant resistance genes can coexist in the same integron and are regulated and expressed by regulatory units (Partridge et al. 2018; Yoon and Jeong 2021). However, whether the same molecular mechanism exists in the strains isolated this study and whether virulence genes, carbapenemase genes, and disinfectant resistance genes coexist in the same mobile element need to be determined in further research.

In paediatric patients, both highly virulent carbapenem-resistant *K. pneumoniae* and highly virulent disinfectant-resistant *K. pneumoniae* have been reported, but there are few reports in the literature of *K. pneumoniae* strains that are resistant to both antibiotics and disinfectants (Candan and Aksöz 2015; Aygun et al. 2019; Surgers et al. 2019). In this study, it was found that all 34 CRKP strains carried virulence genes and disinfectant resistance genes. Although there were differences in the distribution of distinct types of carbapenemase-producing strains, they should be given more attention.

This study had some limitations. First, the data of this retrospective study came from only one hospital and do not represent the characteristics of all Chinese children. Second, this retrospective study was based on data collected from laboratory records, which lack relevant information on the clinical profiles of the children. Therefore, there may be deviations in our assignment of infection and colonization. Third, due to the specificity of the results of the CarbaNP test, it is possible that data from *K. pneumoniae* strains that were negative for the Carba NP et produced carbapenemase may have been missed in this study.

In conclusion, this study presents data on CRKP infection in children, and the finding suggest that *K. pneumoniae* has a higher rate of resistance to carbapenems in paediatric patients. The distribution of antibiotic resistance genes, virulence genes and disinfectant resistance genes of CRKP strains was analysed by whole-genome sequencing. It was found that the CRKP strains were ST11, and all 34 isolates carried both virulence genes and disinfectant resistance genes. The findings strongly suggested that the monitoring of drug resistance, disinfectant resistance and virulence genes of *K. pneumoniae* should be strengthened, especially in the clinical care of children.

## Data Availability

The raw data can be made available to the interested researchers by the authors of this article if requested.

## Abbreviations

CRE: Carbapenem-resistant *Enterobacterales*
ESBLs: Extended-spectrum β-lactamases
CHINET: China antimicrobial surveillance network
CRKP: Carbapenem-resistant *Klebsiella pneumoniae*
cKp: Classic *Klebsiella pneumoniae*
hvKp: Hypervirulent *K. pneumoniae*
MALDI-TOF MS: Matrix-assisted laser desorption ionization time-of-flight mass spectrometry
CLSI: Clinical and Laboratory Standards Institute
CGE: The Center for Genomic Epidemiology
VFDB: Virulence Factors Database of pathogenic bacteria
FDR: False discovery rate

## References

[CR1] Abuzaid AA, Amyes SG (2015). The genetic environment of the antiseptic resistance genes *qacEΔ1* and *cepA* in *Klebsiella pneumoniae*. J Chemother.

[CR2] Aygun F, Aygun FD, Varol F, Durak C, Çokuğraş H, Camcıoğlu Y, Çam H (2019). Infections with carbapenem-resistant gram-negative bacteria are a serious problem among critically ill children: a single-centre retrospective study. Pathogens.

[CR3] Azadpour M, Nowroozi J, Goudarzi GR, Mahmoudvand H (2015). Presence of *qacEΔ1* and c*epA* genes and susceptibility to a hospital biocide in clinical isolates of *Klebsiella pneumoniae* in Iran. Trop Biomed.

[CR4] Beyrouthy R, Dalmasso G, Birer A, Robin F, Bonnet R (2020). Carbapenem resistance conferred by *OXA-48* in *K2-ST86* hypervirulent *Klebsiella pneumoniae*, France. Emerg Infect Dis.

[CR5] Brisse S, Fevre C, Passet V, Issenhuth-Jeanjean S, Tournebize R, Diancourt L, Grimont P (2009). Virulent clones of *Klebsiella pneumoniae*: identification and evolutionary scenario based on genomic and phenotypic characterization. PLoS ONE.

[CR6] Candan ED, Aksöz N (2015). *Klebsiella pneumoniae*: characteristics of carbapenem resistance and virulence factors. Acta Biochim Pol.

[CR7] Catalán-Nájera JC, Garza-Ramos U, Barrios-Camacho H (2017). Hypervirulence and hypermucoviscosity: two different but complementary *Klebsiella spp*. phenotypes?. Virulence.

[CR8] Chen Y, Chen Y (2021). Clinical challenges with hypervirulent *Klebsiella pneumoniae* (hvKP) in China. J Transl Int Med.

[CR9] Chen L, Mathema B, Chavda KD, DeLeo FR, Bonomo RA, Kreiswirth BN (2014). Carbapenemase-producing *Klebsiella pneumoniae*: molecular and genetic decoding. Trends Microbiol.

[CR10] Clegg S, Murphy CN (2016). Epidemiology and virulence of *Klebsiella pneumoniae*. Microbiol Spectr.

[CR11] Clinical and Laboratory Standards Institute (CLSI) (2019). Performance standards for antimicrobial susceptibility testing.

[CR12] Fu L, Wang S, Zhang Z, Yan X, Yang X, Zhang L, Li Y, Wang G, Zhao K, Zhou Y (2019). Co-carrying of *KPC-2, NDM-5, CTX-M-3* and *CTX-M-65* in three plasmids with serotype *O89: H10 Escherichia coli* strain belonging to the *ST2* clone in China. Microb Pathog.

[CR13] Gerlach GF, Clegg S, Allen BL (1989). Identification and characterization of the genes encoding the type 3 and type 1 fimbrial adhesins of *Klebsiella pneumoniae*. J Bacteriol.

[CR14] Gharieb R, Saad M, Khedr M, El Gohary A, Ibrahim H (2022). Occurrence, virulence, carbapenem resistance, susceptibility to disinfectants and public health hazard of *Pseudomonas aeruginosa* isolated from animals, humans and environment in intensive farms. J Appl Microbiol.

[CR15] Han R, Shi Q, Wu S, Yin D, Peng M, Dong D, Zheng Y, Guo Y, Zhang R, Hu F (2020). Dissemination of carbapenemases (*KPC, NDM, OXA-48, IMP*, and *VIM*) among carbapenem-resistant *Enterobacteriaceae* isolated from adult and children patients in China. Front Cell Infect Microbiol.

[CR16] Hansen GT (2021). Continuous evolution: perspective on the epidemiology of carbapenemase resistance among *Enterobacterales* and other gram-negative bacteria. Infect Dis Ther.

[CR17] Ho PL, Li Z, Lai EL, Chiu SS, Cheng VC (2012). Emergence of NDM-1-producing *Enterobacteriaceae* in China. J Antimicrob Chemother.

[CR18] Hsieh PF, Lin TL, Lee CZ, Tsai SF, Wang JT (2008). Serum-induced iron-acquisition systems and *TonB* contribute to virulence in *Klebsiella pneumoniae* causing primary pyogenic liver abscess. J Infect Dis.

[CR19] Hu F, Guo Y, Yang Y, Zheng Y, Wu S, Jiang X, Zhu D, Wang F (2019). Resistance reported from China antimicrobial surveillance network (CHINET) in 2018. Eur J Clin Microbiol Infect Dis.

[CR20] Huang X, Cheng X, Sun P, Tang C, Ni F, Liu G (2018). Characteristics of *NDM-1-*producing Klebsiella pneumoniae *ST234* and *ST1412* isolates spread in a neonatal unit. BMC Microbiol.

[CR21] Kopotsa K, Osei Sekyere J, Mbelle NM (2019). Plasmid evolution in carbapenemase-producing *Enterobacteriaceae*: a review. Ann N Y Acad Sci.

[CR22] Lam MMC, Wyres KL, Judd LM, Wick RR, Jenney A, Brisse S, Holt KE (2018). Tracking key virulence loci encoding aerobactin and salmochelin siderophore synthesis in *Klebsiella pneumoniae*. Genome Med.

[CR23] Lan P, Jiang Y, Zhou J, Yu Y (2021). A global perspective on the convergence of hypervirulence and carbapenem resistance in *Klebsiella pneumoniae*. J Glob Antimicrob Resist.

[CR24] Lawlor MS, O'Connor C, Miller VL (2007). Yersiniabactin is a virulence factor for *Klebsiella pneumoniae* during pulmonary infection. Infect Immun.

[CR25] Li B, Zhao Y, Liu C, Chen Z, Zhou D (2014). Molecular pathogenesis of *Klebsiella pneumoniae*. Future Microbiol.

[CR26] Luo K, Tang J, Qu Y, Yang X, Zhang L, Chen Z, Kuang L, Su M, Mu D (2021). Nosocomial infection by *Klebsiella pneumoniae* among neonates: a molecular epidemiological study. J Hosp Infect.

[CR27] Partridge SR, Kwong SM, Firth N, Jensen SO (2018). Mobile genetic elements associated with antimicrobial resistance. Clin Microbiol Rev.

[CR28] Peng D, Li X, Liu P, Zhou X, Luo M, Su K, Chen S, Zhang Z, He Q, Qiu J, Li Y (2018). Transcriptional regulation of galF by *RcsAB* affects capsular polysaccharide formation in *Klebsiella pneumoniae* NTUH-K2044. Microbiol Res.

[CR29] Pereira RS, Dias VC, Ferreira-Machado AB, Resende JA, Bastos AN, Bastos LQA, Bastos VQA, Bastos RV, Da Silva VL, Diniz CG (2016). Physiological and molecular characteristics of carbapenem resistance in *Klebsiella pneumoniae* and *Enterobacter aerogenes*. J Infect Dev Ctries.

[CR30] Price V, Dunn SJ, Moran RA, Swindells J, McNally A (2022). Whole-genome sequencing enhances existing pathogen and antimicrobial-resistance surveillance schemes within a neonatal unit. Microb Genom.

[CR31] Russo TA, Marr CM (2019). Hypervirulent *Klebsiella pneumoniae*. Clin Microbiol Rev.

[CR32] Russo TA, Olson R, MacDonald U, Beanan J, Davidson BA (2015). Aerobactin, but not yersiniabactin, salmochelin, or enterobactin, enables the growth/survival of hypervirulent (hypermucoviscous) *Klebsiella pneumoniae* ex vivo and in vivo. Infect Immun.

[CR33] Shon AS, Bajwa RP, Russo TA (2013). Hypervirulent (hypermucoviscous) *Klebsiella pneumoniae*: a new and dangerous breed. Virulence.

[CR34] Soto E, Abdelrazek SMR, Basbas C, Duignan PJ, Rios C, Byrne BA (2020). Environmental persistence and disinfectant susceptibility of *Klebsiella pneumoniae* recovered from pinnipeds stranded on the California coast. Vet Microbiol.

[CR35] Surgers L, Boersma P, Girard PM, Homor A, Geneste D, Arlet G, Decré D, Boyd A (2019). Molecular epidemiology of *ESBL*-producing *E. coli* and *K. pneumoniae*: establishing virulence clusters. Infect Drug Resist.

[CR36] Tamma PD, Simner PJ (2018). Phenotypic detection of carbapenemase-producing organisms from clinical isolates. J Clin Microbiol.

[CR37] Van Duin D, Doi Y (2017). The global epidemiology of carbapenemase-producing *Enterobacteriaceae*. Virulence.

[CR38] Wyres KL, Holt KE (2018). *Klebsiella pneumoniae* as a key trafficker of drug resistance genes from environmental to clinically important bacteria. Curr Opin Microbiol.

[CR39] Wyres KL, Lam M, Holt KE (2020). Population genomics of Klebsiella pneumoniae. Nat Rev Microbiol.

[CR40] Yang Y, Liu JH, Hu XX, Zhang W, Nie TY, Yang XY, Wang XK, Li CR, You XF (2020). Clinical and microbiological characteristics of hypervirulent *Klebsiella pneumoniae* (hvKp) in a hospital from North China. J Infect Dev Ctries.

[CR41] Yoon EJ, Jeong SH (2021). Mobile carbapenemase genes in *Pseudomonas aeruginosa*. Front Microbiol.

[CR42] Yu J, Wang Y, Chen Z, Zhu X, Tian L, Li L, Sun Z (2017). Outbreak of nosocomial *NDM-1-*producing *Klebsiella pneumoniae ST1419* in a neonatal unit. J Glob Antimicrob Resist.

[CR43] Zhang Y, Wang Q, Yin Y, Chen H, Jin L, Gu B, Xie L, Yang C, Ma X, Li H, Li W, Zhang X, Liao K, Man S, Wang S, Wen H, Li B, Guo Z, Tian J, Pei F, Liu L, Zhang L, Zou C, Hu T, Cai J, Yang H, Huang J, Jia X, Huang W, Cao B, Wang H (2018). Epidemiology of carbapenem-resistant *Enterobacteriaceae* infections: report from the China CRE network. Antimicrob Agents Chemother.

[CR44] Zheng B, Xu H, Yu X, Lv T, Jiang X, Cheng H, Zhang J, Chen Y, Huang C, Xiao Y (2018). Identification and genomic characterization of a *KPC-2-, NDM-1-* and *NDM-5*-producing *Klebsiella michiganensis* isolate. J Antimicrob Chemother.

